# Blessing in disguise: when head trauma solves the riddle of carbonic anhydrase II deficiency

**DOI:** 10.1016/j.radcr.2021.12.004

**Published:** 2022-01-06

**Authors:** Yazan O. Al Zu'bi, Ahmed H. Al Sharie, Waed Dwairi, Eyad Altamimi

**Affiliations:** a Faculty of Medicine, Jordan University of Science and Technology, Irbid 22110, Jordan; b Pediatric Department, Faculty of Medicine, Jordan University of Science and Technology, P.O. Box. 3030, Irbid 22110, Jordan

**Keywords:** Carbonic anhydrase II deficiency, CA II, Osteopetrosis, Intracerebral calcifications, Renal tubular acidosis

## Abstract

Carbonic anhydrase II deficiency is a rare autosomal recessive disorder with a classical triad of renal tubular acidosis, intracerebral calcifications and osteopetrosis. We present a case of a 6-year and 4-months old male patient presented to our pediatric gastroenterology outpatients’ clinic with parental concern of poor growth. The patient is a known case of unexplained global developmental delay, recurrent fractures and constipation since birth. As a result of the patient's hyperactivity, he hit his head with the clinic's door resulting in a cut wound. Brain computed tomography scan showed abnormal symmetrical calcifications seen in both basal ganglia, thalami and subcortical white matter associated with increased bone density of the skull and upper cervical spine reassembling osteopetrosis. The suspicion of carbonic anhydrase II deficiency was confirmed by arterial blood gases revealing a marked metabolic acidosis fulfilling the diagnostic triad. The patient was discharged on sodium bicarbonate therapy, lactulose and vitamin D_3_ supplements and has been followed up regularly.

## Introduction

Carbonic anhydrase II (CA II) deficiency is a rare autosomal recessive inborn error of metabolism with a pathognomonic triad of renal tubular acidosis, intracerebral calcifications and osteopetrosis [1]. This disease entity was first recognized in 1972 by three reports of independent families in America, Belgium and France [2]. Other associated clinical features include: cranial nerve compression, developmental delay, dental abnormalities, short stature with wide spectrum of cognitive deficits ranges from simple learning disabilities to mental retardation [3]. A possible correlation has been reported between patient's ethnicity and the severity of mental retardation in which Japanese and Arabian groups exhibited a more severe form of developmental delay compared to the American population [1]. In contrast to malignant osteopetrosis, CA II deficiency exhibits an indolent path [1]. Currently, there is no specific treatment for CA II deficiency apart from correcting electrolyte and acid-base imbalance. The role of bone marrow transplant in such clinical setting is poorly studied in terms of eligibility, risk factors, and long term sequala. According to the available evidence, bone marrow transplant can resolve osteopetrosis and intracerebral calcification with little effect regarding renal tubular acidosis [4,5].

## Case presentation

A 6-year and 4-months old male Arab patient presented to our pediatric gastroenterology outpatients’ clinic with parental concern of poor growth. The patient is a known case of unexplained global developmental delay, recurrent fractures and constipation since birth. The latter being investigated 3 years ago with laboratory workup and upper gastrointestinal endoscopy, in which no significant findings were yielded. Both his weight and height were below the third centile. His complaint was addressed using laxatives resulting in a non-satisfactory response. The patient is a product of a normal vaginal delivery weighted 2.6 Kg at birth with a history of delayed meconium passage. In the current visit, the mother reported that he has poor appetite and constipation, passing stool every 7 to 9 days, the stool is hard in consistency with no mucous or blood associated. The patient is a picky eater with small meals as he doesn't consume chicken, meat or yogurt. His main nutritional sources are chocolates, chips, jelly and juices besides fruits and vegetables. Apart from this, the systematic review of symptoms and the physical examination were unremarkable.

The patient is highly inattentive and hyperactive evaluated by the psychiatry team for attention-deficit/hyperactivity disorder. Upon leaving the clinic, the patient hit his head with the door resulting in a cut wound which required stitching. Vital signs and neurological exam were normal. Brain computed tomography (CT) scan could not be performed due to patient's hyperactivity and poor cooperativity. He was admitted for observation. During his admission, basic laboratory investigations were ordered including: complete blood count, liver function test, kidney function test, serum electrolytes, urine analysis, inflammatory markers, ferritin, parathyroid hormone levels, vitamin B12, vitamin D and celiac serology profile. All were within normal levels except for hypermagnesemia, hyperphosphatemia, hyperchloremia, increased urine chloride levels and negative celiac serology profile.

**Fig. 1 –:**
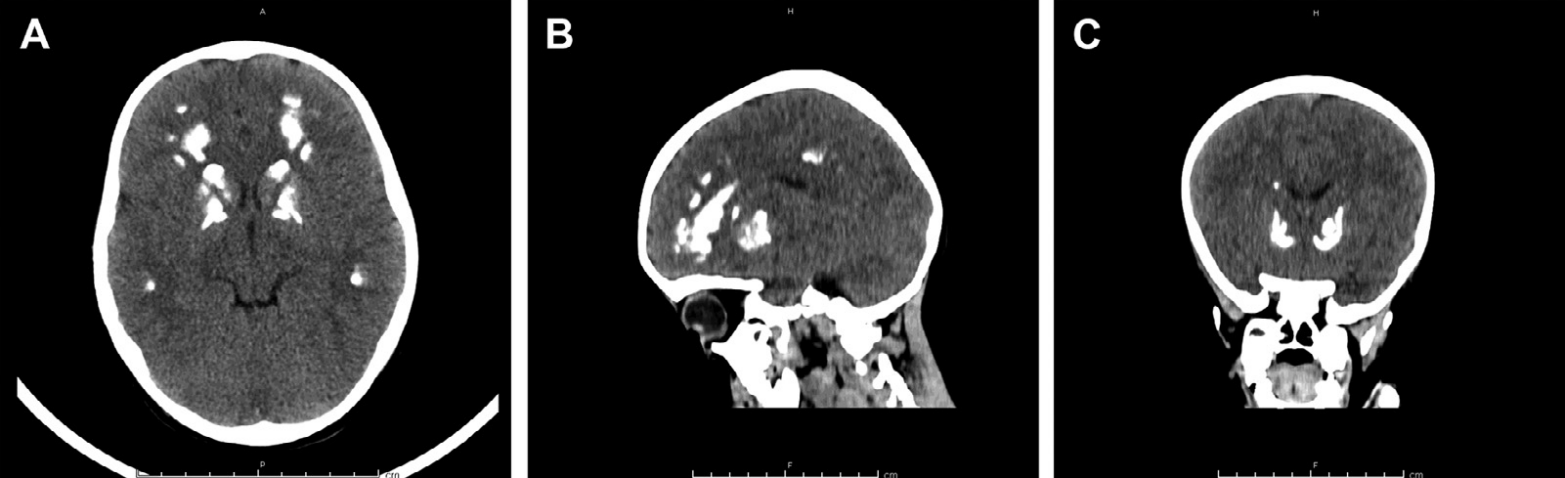
Brain computed tomography (CT) scan showed abnormal symmetrical calcifications seen in the both basal ganglia, thalami and subcortical white matter (A-C) associated with increased bone density of the skull and upper cervical spine (B and C).

**Fig. 2 –:**
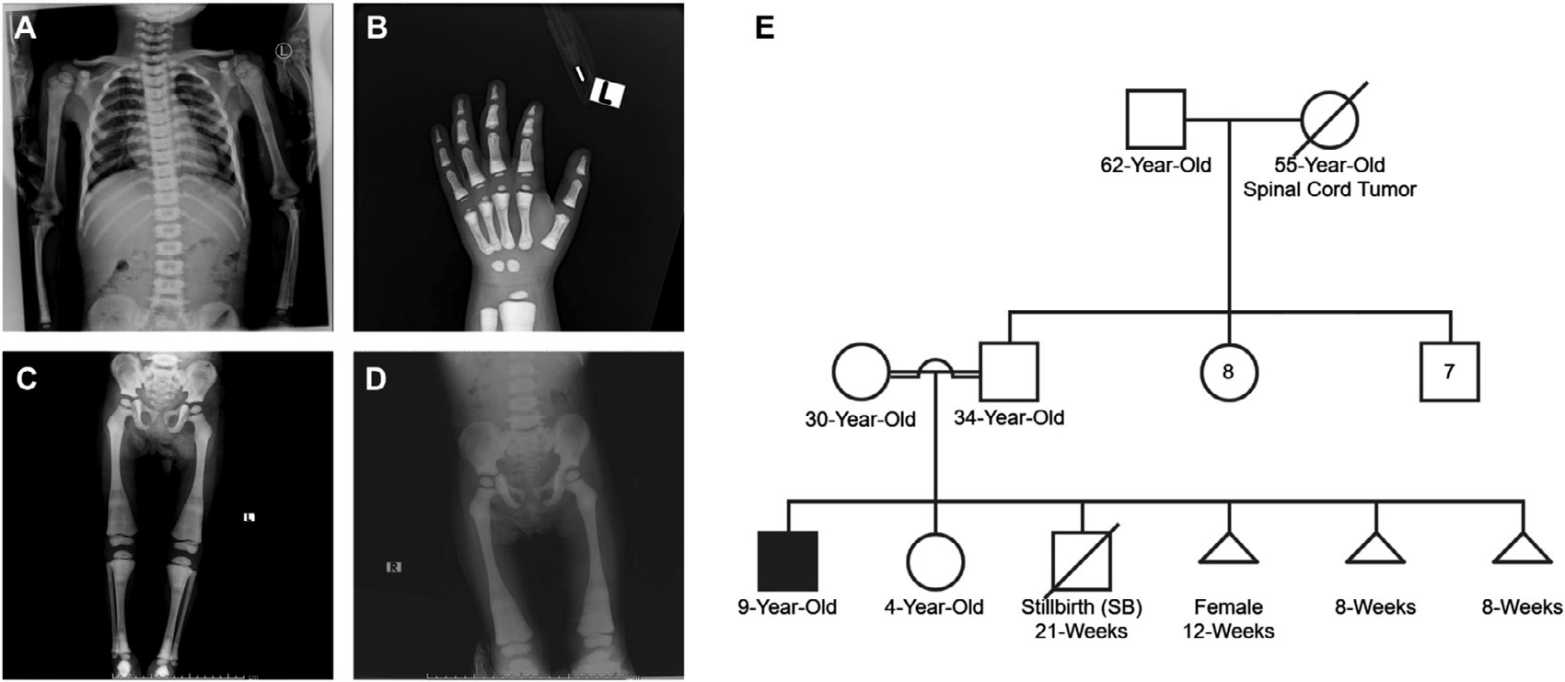
Bone age assessment and skeletal survey studies revealing osteopetrotic changes consistent with carbonic anhydrase II (CA II) deficiency (A-D). Pedigree chart of the family generated during 2021 highlighting the affected male with CA II deficiency confirmed by whole exome sequencing (E).

On the next day, the mother reported excessive sleepiness and hypoactivity, hence a brain CT scan was performed (Fig. 1). The scan showed abnormal symmetrical calcifications seen in both basal ganglia, thalami and subcortical white matter associated with increased bone density of the skull and upper cervical spine. The current findings with previously observed changes in X-rays are highly suggestive of osteopetrosis seen with carbonic anhydrase II deficiency. Consequently, arterial blood gases (ABGs), bone age assessment and skeletal survey (Fig. 2A-D) were performed. ABGs shows a metabolic acidosis with a pH of 7.24, *P*CO_2_ of 25 mm Hg, HCO_3_^−^ of 10.5 mmol/L, *P*O_2_ of 161.60 mm Hg, oxygen saturation of 98.9% with a base excess of -14.9 mmol/L. The current clinical findings including renal tubular acidosis, intracranial calcification and osteopetrosis suggests CA II deficiency. Whole exome sequencing confirms the diagnosis of CA II deficiency with homozygous mutation (CA2 NM_000067.3:c.232+1G>A) inherited in an autosomal recessive manner. The Substitution single nucleotide mutation at the splicing junction at 8q21.2 produces an abnormal splicing effect, which is expected to cause a loss of normal protein function via nonsense-mediated mRNA decay. The mutation is previously reported as the classical mutation in the Arabian Peninsula. Such mutation has been also previously reported to be a pathogenic mutation with a ClinVar ID: VCV000288909.

The diagnosis is further supported by the consanguinity between the patient's parents as they are cousins (Third-degree relatives). During the hospitalization period, he developed a single seizure attack characterized as tonic seizure associated with up rolling of the eyes and cyanosis which was relieved without medications. Electroencephalography and brain magnetic resonance imaging (MRI) were performed. Brain MRI revealed multiple area of blooming artifacts in the subcortical white matter of both cerebral hemisphere and in both basal ganglia corresponding to areas of calcifications previously seen in the CT scan. The patient was discharged on the following medications including sodium bicarbonate (1000 mg, Q8, Oral), lactulose (15 mL, Q12), vitamin D_3_ supplement (50,000 IU once a week) and risperidone (1 mg, Q24 at bedtime, oral). The patient was followed up in the pediatric neurology outpatient's clinic in which carbamazepine (100 mg, Q8) was started according to Electroencephalography and brain MRI findings. Table 1 illustrates the ABGs findings during admission and future follow ups after medical therapy.

**Table 1 –:** Arterial blood gases (ABGs) findings during admission and follow-ups.

ABGs parameters	Day 6	Day 7	Day 12	1-mo follow-up	4-mo follow-up
pH	7.24	7.25	7.23	7.24	7.24
*P*CO_2_	25.00	43.40	48.40	50.00	48.80
HCO_3_^−^	10.50	18.90	20.3	21.00	20.3
*P*O_2_	161.60	34.70	28.30	31.50	21.70
O_2_ saturation	98.90	54.40	39.70	47.10	*
Base excess	-14.90	-7.90	-7.20	-6.60	-7.1

Days were calculated after admission.

⁎ Not available.

## Discussion

Carbonic anhydrases (CAs) are a metalloenzyme superfamily that was firstly discovered back in 1933, they primarily catalyze the reversible hydration of carbon dioxide (CO_2_) into carbonic acid (H_2_CO_3_) [6]. CAs have been classified into six major categories including alpha, beta, gamma, delta, zeta, and eta; with alpha being exclusively found in mammals including humans [6,7]. There have been 15 diverse human CA isoforms identified in the literature, 12 of which are enzymatically dynamic, while the remaining three are CA-related proteins. Albeit the similarities; they vary in their molecular structure, location within the cell, susceptibility to inhibitors and their placement across different tissues and organs [8]. Specifically, CA II is the most broadly studied of all human carbonic anhydrases, it is a cytoplasmic monomeric protein with a mass of 29 kDa embracing 259 amino acids encoded by the *CA2* gene located at q22 on chromosome number 8 [7,9,10]. CA II is found ubiquitously, but majorly inside red blood cells, the alimentary tract, kidneys, eyes, lungs, osteoclasts, testis, and brain [8]. Particularly, the corner stone of the CA II active site is the zinc metal which is found in a distorted tetrahedrally coordinated matter in accordance to the imidazole groups of the three histidine residues [11]. The catalytic process occurs by the nucleophilic attack of the zinc-hydroxide on the CO_2_ molecule in a ping pong two-step mechanism, resulting in the production of a bicarbonate (HCO_3_^−^) which is latterly shifted away through a water molecule (H_2_O) [12].

CA II has been observed to be associated with various biological processes inside the human body, its presence within the cells of the proximal convoluted tubules participates in sodium reabsorption and hydrogen secretion besides the sodium/proton exchanger isoform 3 (NHE3) [13], proton secretion is also affected via a functional interaction with anion exchanger 1, in addition to that it plays a major role augmented with aquaporin-1 increasing the water movement resulting in urine concentration, hence a deficiency in the enzyme would result in failure of generating protons which are essential for the HCO_3_^−^ reabsorption and apical protons secretion [14], [15], [16]. CA II is assuming a significant part of the renal physiology with over 95% commitment to the nephron functionalities [9,14]. It is also implicated in the process of bone resorption by maintaining an acidic microenvironment during the process of bone remodeling [14,17].

In addition to acid-base balance and fluid electrolyte homeostasis, CA II plays a critical homeostatic role in aqueous humor and cerebrospinal fluid, gastric acidity, pancreatic enzymes secretion along with urogenesis, lipogenesis, and gluconeogenesis [18,19]. Therefore, CA II deficiency is a multisystem disorder. It was firstly described in three independent kindreds in 1972 [20], [21], [22]. CA II deficiency syndrome is otherwise known as marble brain disease or Guibaud-Vainsel syndrome which is a rare autosomal recessive inborn error of metabolism, classically presents as a triad of osteopetrosis, cerebral calcification and renal tubular acidosis which contains both a proximal and a distal component, with one of which being dominant in any particular case [17,23,24]. However, several other complications may also arise including growth failure, developmental delay, and mental retardation. Particularly, osteopetrosis can result into multiple frequent fractures, cranial nerve palsies due to compression effect resulting into blindness and deafness along with dental malocclusions [17,25]. This is consistent with our described case showing the classical picture of CA II deficiency with a chief complaint of failure to thrive, and evident renal tubular acidosis besides osteopetrotic changes, along with intra-cranial calcification on CT scan and global developmental delay. Conversely Hu et al. reported an atypical presentation with no evidence of renal tubular acidosis [26]. Palmo et al. on the other hand reported a case of CA II deficiency consequently resulting into a severe obstructive sleep apnea [3].

Patients with CA II deficiency have to be followed carefully to prevent any critical complications such as cranial nerve entrapment, stone-related obstructive uropathy, in addition to respiratory arrest [23]. The long-term effects are mainly neurological in origin; in a report of 35 children which were followed investigators reported effects of progressive osteopetrosis and cerebral calcification, blindness, anemia, and resultant erythropoiesis due to bone marrow involvement, two patients got married with children free of the disease [27]. Hematological complications are only visualized in severe infantile form of osteopetrosis, consequently resulting into anemia, leukopenia and thrombocytopenia, yet has no role in CA II deficiency [23]. Bone marrow transplantation could be considered beneficial in the resolution of osteopetrosis histologically and radiologically, along with impeding cerebral calcifications. Nonetheless it didn't show any improvement regarding the renal tubular acidosis and mental retardation as reported by McMahon et al. in a case of two children from Irland [4]. However, for now the keystone of treatment remains an alkali therapy [25].

Hitherto the disease entity is more predominant in the Middle east and Mediterranean regions, due to the higher consanguinity rates compared to the western populations which is a common feature in families with CA II deficiency [23,25]. Likewise, within our case medically free parents were first-degree cousins. It has been described in multiple ethnic groups as well including Caucasian, Hispanic, African, let alone Arabians which contributes to 70% of the cases [28,29]. The most frequently detected mutation is a loss of the splice donor site at the 5 prime end of intron 2 which is colloquially known as the Arabic mutation [29]. Yet the prevailing mutation in Hispanics is a single base mutation in exon 7 resulting into a frameshift changing the subsequent 12 amino acids [26].

## Conclusion

Herein, we report a case of a CA II deficiency which was diagnosed after a head trauma in the clinic, the mystery behind the patient's suffering could have been further delayed without the role of serendipity and luck as a phenomenon which cannot be disregarded in our case. Although, CA II deficiency is characterized as an ultrarare disorder, the majority of affected individuals’ descent form the Arabian population, rendering the importance of early recognition, diagnosis and treatment to avoid long term unreversible manifestations, especially when dealing with Mediterranean patients.

## Availability of data and materials

Not applicable.

## Ethics approval and consent to participate

This report has been conducted and written in accordance with the ongoing regulations for case reports and case series in the king Abdullah university hospital (KAUH). Case reports are exempted from institutional ethical approval by the institutional review board (IRB). Written informed consent was obtained from the patient for publication of this report and any associated images. A copy of the consent is available upon request.
